# Pediatric interfacility transport effects on mortality and length of stay

**DOI:** 10.1007/s12519-021-00445-w

**Published:** 2021-07-28

**Authors:** Rod M. Shinozaki, Andreas Schwingshackl, Neeraj Srivastava, Tristan Grogan, Robert B. Kelly

**Affiliations:** 1 David Geffen School of Medicine at UCLA, Los Angeles, CA USA; 2 University of California, Irvine, Irvine, CA USA; 3 Children’s Hospital of Orange County, 1201 West La Veta Avenue, Orange, CA 92868 USA

**Keywords:** Helicopter, Hospital length of stay, Pediatric critical care, Pediatric intensive care unit, Transport medicine

## Abstract

**Background:**

We aimed to evaluate the effects of interfacility pediatric critical care transport response time, physician presence during transport, and mode of transport on mortality and length of stay (LOS) among pediatric patients. We hypothesized that a shorter response time and helicopter transports, but not physician presence, are associated with lower mortality and a shorter LOS.

**Methods:**

Retrospective, single-center, cohort study of 841 patients (< 19 years) transported to a quaternary pediatric intensive care unit and cardiovascular intensive care unit between 2014 and 2018 utilizing patient charts and transport records. Multivariate linear and logistic regression analyses adjusted for age, diagnosis, mode of transport, response time, stabilization time, return duration, mortality risk (pediatric index of mortality-2 and pediatric risk of mortality-3), and inotrope, vasopressor, or mechanical ventilation presence on admission.

**Results:**

Four hundred and twenty-eight (50.9%) patients were transported by helicopter, and 413 (49.1%) were transported by ambulance. Physicians accompanied 239 (28.4%) transports. The median response time was 2.0 (interquartile range 1.4–2.9) hours. Although physician presence increased the median response time by 0.26 hours (*P* = 0.020), neither physician presence nor response time significantly affected mortality, ICU length of stay (ILOS) or hospital length of stay (HLOS). Helicopter transports were not significantly associated with mortality or ILOS, but were associated with a longer HLOS (3.24 days, 95% confidence interval 0.59–5.90) than ambulance transports (*P* = 0.017).

**Conclusions:**

These results suggest response time and physician presence do not significantly affect mortality or LOS. This may reflect the quality of pre-transport care and medical control communication. Helicopter transports were only associated with a longer HLOS. Our analysis provides a framework for examining transport workforce needs and associated costs.

## Introduction

Currently, no evidence-based pediatric guidelines exist that define the requirement for physician presence on interfacility transports, the ideal mode of transportation, or limitations of mobilization and travel times to and from outside facilities due, in part, to the paucity of studies in this field [1–4]. However, several reports suggest that the outcomes of children improve when they are transported by specialty pediatric transport teams rather than basic emergency medical services [5–7], as evidenced by improved mortality rates and decreased frequency of adverse events during transport [6, 8–10]. Non-physician transport team members have shown strong potential in providing procedural interventions, such as high successful intubation rates of 95–100% [11, 12]. One study comparing respiratory therapists to resident physicians on a transport team showed higher success rates for endotracheal intubation among respiratory therapists (92% vs. 77%) [13]. To date, only one study has compared specialty pediatric transport teams with and without a physician, and no differences in mortality were seen when adjusted for the severity of illness of the transported patients [12].

Furthermore, when comparing air versus ground transports, studies have suggested that helicopters are faster at transporting patients than ambulances [14] and are associated with improved survival in adult [15] and pediatric trauma patients [16–18]. Only one adult study showed that transport time intervals are independently associated with intensive care unit (ICU) length of stay (ILOS) and hospital length of stay (HLOS) [19], and one neonatal study on premature infants showed that faster response times were not associated with improved outcomes [20].

Our aim was to investigate whether an association exists between (1) interfacility response time; (2) mode of transport; and (3) physician presence during the transport of critically ill children and clinically relevant outcomes, including (1) mortality; (2) ILOS; and (3) HLOS. We hypothesized that a shorter response time and helicopter transports, but not physician presence, are associated with lower mortality and a shorter LOS after adjusting for relevant risk factors.

## Methods

### Setting

The UCLA Mattel Children’s Hospital pediatric ICU (PICU) is a 24-bed quaternary unit affiliated with the University of California, Los Angeles, which also cares for post-operative cardiovascular surgical patients. Our pediatric critical care transport (PCCT) team includes, at a minimum, a respiratory therapist and a pediatric critical care nurse. Only if the PICU team deems it necessary given the patient’s perceived clinical condition will the on-call (home or in-house) physician be activated. The physician team includes PICU and neonatal ICU (NICU) attendings, pediatric critical care and neonatal–perinatal medicine fellows, and emergency medicine resident physicians. Respiratory therapists are required to have a number of competencies within the adult ICU, PICU, NICU, and emergency department. All members complete air flight safety classes and equipment workshops. Our PCCT team nurses are highly trained with a minimum of 3 years of experience as bedside PICU and/or NICU nurses before being trained to go on transports. PCCT team physicians are not required to be on site at all times, but available within 30 minutes. The PCCT team’s intake area extends throughout California and Nevada, but most transported patients are within a heavily trafficked metropolitan region. Each year the PCCT team performs an average of over 350 pediatric and neonatal critical care transports by ground and air. REACH Air Medical Services (Santa Rosa, CA) is the preferred helicopter provider for UCLA Mattel Children’s Hospital, but our own UCLA PCCT team members accompany REACH crewmembers as the primary care providers.

### Data collection

**Fig. 1:**
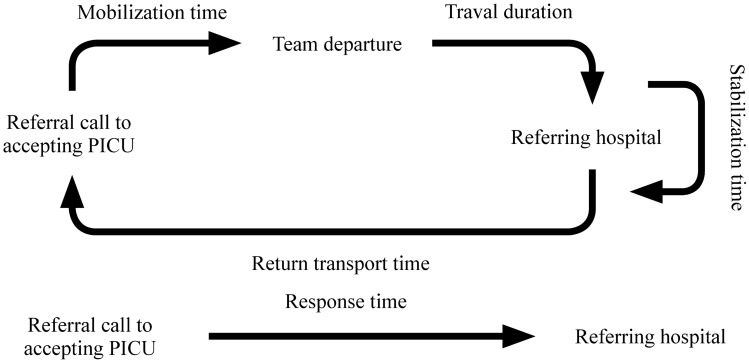
Depiction of a typical interfacility transport and associated time intervals. *PICU* pediatric intensive care unit

The UCLA Institutional Review Board approved this study and waived informed consent. A chart review of all PCCTs between January 2014 and August 2018 was performed. A patient met inclusion criteria if they were transported by our PCCT team to the PICU at UCLA and were 18 years of age or younger. Exclusion criteria included patients who were not transported to the PICU at UCLA, were transported by fixed-wing aircraft, were patients greater than 18 years of age, or were intra-facility transports. Original data variables of age, diagnosis, mode of transport, physician presence during transport, and transport team departure time were obtained from the UCLA Mattel Children’s Hospital’s transport database. These data elements are entered by the critical care transport nurse after each transport. We defined the following transport time intervals as: mobilization time = time from referral call to team departure; travel duration = time from accepting hospital to the referring hospital; response time = mobilization time + travel duration; stabilization time = time spent preparing the patient to leave; return transport time = time spent traveling back (Fig. 1). Through UCLA’s Clinical and Translational Science Institute’s informatics program, we reviewed each patient’s electronic health record to extract information on inotrope, vasopressor, and mechanical ventilation requirements on admission. Registry Partners (Burlington, NC), an independent data extraction group contracted with UCLA, provided pediatric index of mortality-2 (PIM-2) [21], pediatric risk of mortality-3 (PRISM-3) [22], and PRISM-3 probability of death (POD) [23] scores for each transported patient admitted to the PICU. To provide consistency, a single investigator (RMS) coordinated or performed all data abstraction.

### Statistical methods

Patient characteristics and study variables were summarized using the medians [interquartile range (IQR)] for continuous variables or frequency (%) for categorical variables. We compared patient, transport, and outcome variables between physician presence and mode of transportation using the Chi-square test or Student’s *t* test, as appropriate. Finally, to determine if physician presence, response time, or air transport were associated with our predefined study outcomes (mortality, ILOS, and HLOS) after adjusting for patient morbidity (PIM-2 and PRISM-3 scores) and other patient characteristics, we ran multivariable logistic or linear regression models. To assess outcomes (mortality, ILOS, and HLOS) between diagnostic categories, we used trauma patients as the referent category, since this group was moderately sized and had the lowest LOS of all 10 diagnostic groups. We ran complete case analyses for these models, and seven patients were excluded due to missing stabilization and/or return times. These seven patients did not appear characteristically different than the other patients, therefore the assumption of these to be missing completely at random seemed reasonable, and we proceeded with the analyses. From the models, we extracted the relevant odds ratios with 95% confidence intervals (CIs) or effect sizes with 95% CIs. A propensity score modeling approach was also run as a sensitivity analysis for physician presence and air versus ground transports. Statistical analyses were performed using SPSS V25 (IBM Corp., Armonk, NY) or R V3.5.1 (www.r-project.org, Vienna, Austria). *P* values < 0.05 were considered statistically significant. Standard mortality ratios (SMR) were calculated by observed mortality within our ICU divided by expected mortality from PRISM-3 POD.

## Results

**Fig. 2:**
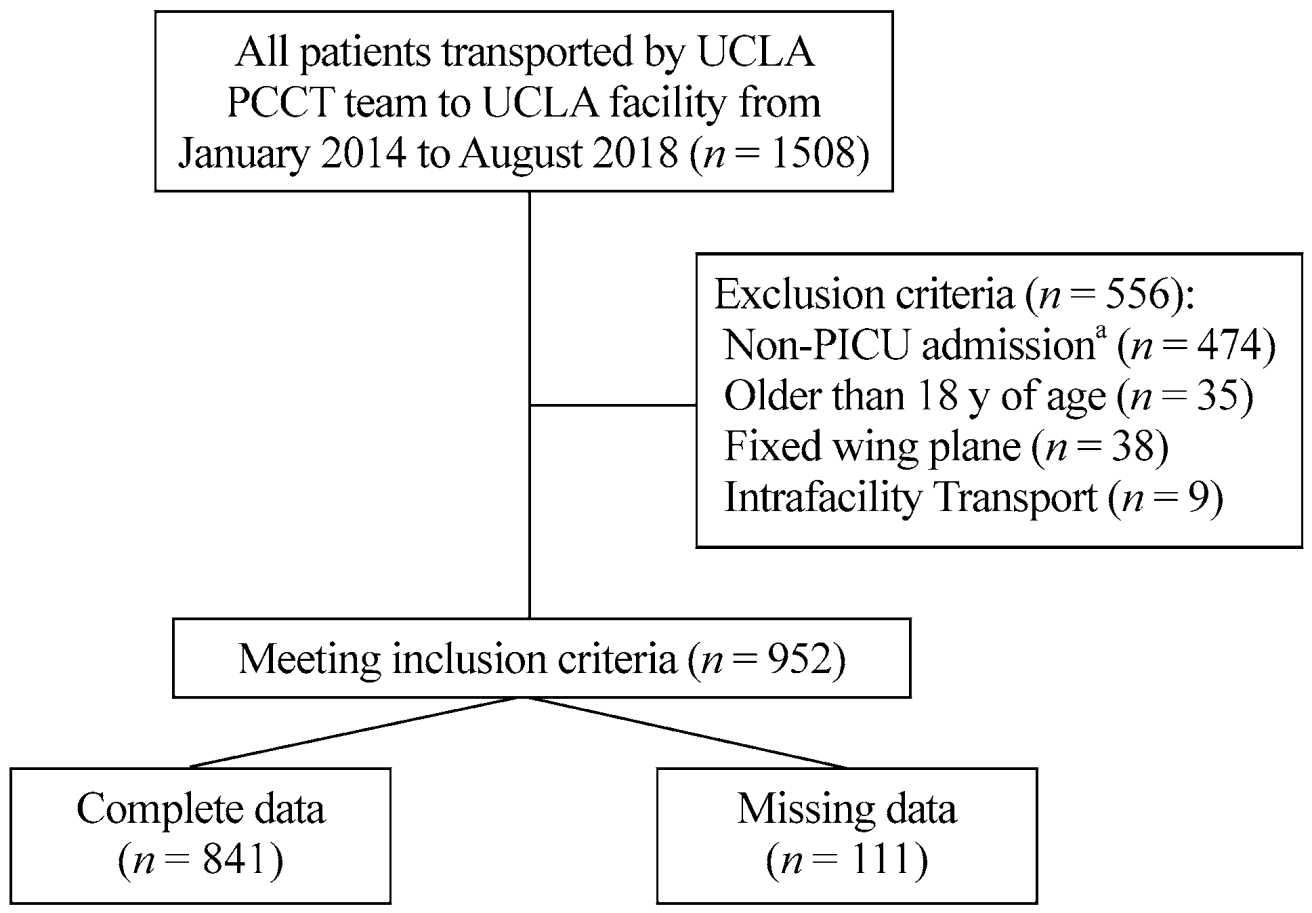
Depiction of study population analyzed. *PCCT* pediatric critical care transport, *PICU* pediatric intensive care unit, *NICU* neonatal intensive care unit. ^a^NICU, inpatient wards, step-down off-site PICU, emergency room

Between January 2014 and August 2018, 1508 pediatric patients were transported to UCLA by our PCCT team. After excluding 556 patients due to non-PICU transports, fixed-wing transports, transports of patients greater than 18 years of age, and intra-facility transports, 952 patients met inclusion criteria. However, due to missing data points, ultimately 841 patient transports were analyzed (Fig. 2). The median age of the transported PICU patients was 4.91 (IQR 0.99–12.96) years. The most common diagnoses were categorized as respiratory (252, 30.0%), neurological (224, 26.6%), and cardiovascular (99, 11.8%). The median PIM-2 score was – 4.56 (IQR – 4.83 to 3.33), and the median PRISM-3 score was 2 (IQR 0–6). On admission, 159 (18.9%) patients were receiving inotrope or vasopressor infusions, and 174 (20.7%) patients were mechanically ventilated. Helicopter transports were utilized for 428 (50.9%) patients, and ambulance transports were utilized for 413 (49.1%). Physicians were present on 239 (28.4%) transports. The median response time was 2.0 (IQR 1.4–2.9) hours, and the median stabilization time was 0.67 (IQR 0.45–0.97) hours. The all-cause mortality rate during the first 48 hours after admission was 1.5% (13 patients) and 6.4% (54 patients) during hospitalization. The overall SMR was 1.64. If a physician was present on the transport, the SMR was 1.60, compared to 1.73 when a physician was not present (*P* = 0.812). The SMR for helicopter transports was 1.77 compared to 1.44 for ground transports (*P* = 0.369). The median ILOS was 2.7 (IQR 1.4–7.0) days, and the median HLOS was 5.0 (IQR 2.0–13.3) days (Table 1).

**Table 1:** Summary of demographics and outcomes (*n* = 841)

Patient characteristics	Values
Median age (y), median (IQR)	4.91 (0.99–12.96)
Diagnosis, *n* (%)	
Respiratory	252 (30.0)
Neurologic	224 (26.6)
Cardiovascular	99 (11.8)
Sepsis	61 (7.3)
Gastrointestinal	49 (5.8)
Trauma	46 (5.5)
Metabolic	44 (5.2)
Other	27 (3.2)
Hematology/oncology	21 (2.5)
Renal	18 (2.1)
Median PIM-2, median (IQR)	− 4.56 (− 4.83 to − 3.33)
Median PRISM-3, median (IQR)	2 (0–6)
Median PRISM-3 POD, median (IQR)	0.51 (0.30–1.30)
Inotrope/vasopressor drip on admission, *n* (%)	159 (18.9)
Ventilated on admission, *n* (%)	174 (20.7)
Transport characteristics	
Helicopter (vs. ground), *n* (%)	428 (50.9)
Emergent transport (vs. urgent), *n* (%)	308 (36.6)
Physician present, *n* (%)	239 (28.4)
Median mobilization time (h), median (IQR)	1.33 (0.88–1.98)
Median stabilization time (h), median (IQR)	0.67 (0.45–0.97)
Median return time (h), median (IQR)	0.58 (0.35–0.88)
Median stabilization + return time (h), median (IQR)	1.33 (0.99–1.83)
Median response time (h), median (IQR)	2.0 (1.4–2.9)
Outcomes	
Mortality (within 48 h of hospital admission), *n* (%)	13 (1.5)
Mortality (during hospital admission), *n* (%)	54 (6.4)
Median hospital LOS (d), median (IQR)	5.0 (2.0–13.3)
Median ICU LOS (d), median (IQR)	2.7 (1.4–7.0)

Due to some missing time interval data, time intervals are not additive. *PIM-2* pediatric index of mortality-2, *PRISM-3* pediatric risk of mortality-3, *POD* probability of death, *LOS* length of stay, *ICU* intensive care unit, *IQR* 25–75th interquartile range

Univariate analysis revealed that physician presence and helicopter transports were both significantly associated with worse PIM-2 and PRISM-3 scores (Tables 2 and 3). The median response time when physicians were present was 2.18 hours compared to 1.92 hours when no physician was present, resulting in a difference of 0.26 hours (*P* = 0.020). However, physician presence and response time were not significantly associated with our outcomes of interest (mortality, ILOS and HLOS) when adjusting for age, diagnosis, mode of transport, response time, stabilization time, return duration, mortality risk (PIM-2 and PRISM-3), and inotrope, vasopressor or mechanical ventilation presence on admission (Table 4). Compared to ground transports, helicopter transports resulted in an average increase in HLOS of 3.24 (95% CI 0.59–5.90; *P* = 0.017) days, but not in ILOS (average 1.68 days; 95% CI – 0.11 to 3.47; *P* = 0.066). As a sensitivity analysis, we elected to perform a propensity score matched analysis with the same variables to confirm our multivariable model estimates for both the effect of physician presence and helicopter transports on our three outcomes of interest (mortality, ILOS, and HLOS). In general, the multivariable and propensity score models produced similar estimates and *P* values (results not shown).

**Table 2:** Comparison between physician presence and patient characteristics

Patient characteristics	Physician presence		
	No (*n* = 602)	Yes (*n* = 239)	*P*
Median age (y), median (IQR)	5.44 (1.18–13.13)	3.07 (0.59–12.07)	0.030
Diagnosis, *n* (%)			< 0.001
Respiratory	164 (27.2)	88 (36.8)	
Neurologic	183 (30.4)	41 (17.2)	
Cardiovascular	48 (8.0)	51 (21.3)	
Sepsis	43 (7.1)	18 (7.5)	
Gastrointestinal	36 (6.0)	13 (5.4)	
Trauma	36 (6.0)	10 (4.2)	
Metabolic	38 (6.3)	6 (2.5)	
Other	20 (3.3)	7 (2.9)	
Hematology/oncology	17 (2.8)	4 (1.7)	
Renal	17 (2.8)	1 (0.4)	
Median PIM-2, median (IQR)	− 4.63 (− 4.86 to − 4.28)	− 3.41 (− 4.61 to − 2.74)	< 0.001
Median PRISM-3, median (IQR)	0.00 (0.00–4.00)	5.00 (0.00–10.00)	< 0.001
Median PRISM-3 POD, median (IQR)	0.49 (0.30–0.90)	1.05 (0.51–3.91)	< 0.001
Inotrope/vasopressor drip on admission, *n* (%)	64 (10.6)	95 (39.7)	< 0.001
Ventilated on admission, *n* (%)	45 (7.5)	129 (54.0)	< 0.001
Transport characteristics			
Helicopter (vs. ground), *n* (%)	271 (45.0)	157 (65.7)	< 0.001
Emergent transport (vs. urgent), *n* (%)	178 (29.6)	130 (54.4)	< 0.001
Physician present, *n* (%)	0 (0)	239 (100)	–
Median mobilization time (h), median (IQR)	1.23 (0.80–1.88)	1.47 (1.08–2.28)	< 0.001
Median stabilization time (h), median (IQR)	0.58 (0.42–0.80)	1.00 (0.75–1.42)	< 0.001
Median return time (h), median (IQR)	0.53 (0.33–0.83)	0.63 (0.40–1.00)	0.023
Median stabilization + return time (h), median (IQR)	1.17 (0.92–1.57)	1.73 (1.30–2.27)	< 0.001
Median response time (h), median (IQR)	1.92 (1.26–2.78)	2.18 (1.58−3.25)	0.020
Outcomes			
Mortality (within 48 h of hospital admission), *n* (%)	4 (0.7)	9 (3.8)	0.001
Mortality (during hospital admission), *n* (%)	20 (3.3)	34 (14.2)	< 0.001
Median hospital LOS (d), median (IQR)	4.25 (1.75–9.64)	9.57 (3.42−22.10)	< 0.001
Median ICU LOS (d), median (IQR)	2.00 (1.13–4.76)	6.76 (2.40–17.29)	< 0.001

Due to some missing time interval data, time intervals are not additive. *PIM-2* pediatric index of mortality-2, *PRISM-3* pediatric risk of mortality-3, *POD* probability of death, *LOS* length of stay, *ICU* intensive care unit, *IQR* 25–75th interquartile range. “–” none

**Table 3:** Comparison between mode of transport and patient characteristics

Patient characteristics	Mode of transport		
	Ground (*n* = 413)	Air (*n* = 428)	*P*
Median age (y), median (IQR)	5.86 (1.22–13.46)	3.91 (0.67–12.43)	0.034
Diagnosis, *n* (%)			0.057
Respiratory	121 (29.3)	131 (30.6)	
Neurologic	113 (27.4)	111 (25.9)	
Cardiovascular	41 (9.9)	58 (13.6)	
Sepsis	33 (8.0)	28 (6.5)	
Gastrointestinal	21 (5.1)	28 (6.5)	
Trauma	19 (4.6)	27 (6.3)	
Metabolic	22 (5.3)	22 (5.1)	
Other	19 (4.6)	8 (1.9)	
Hematology/oncology	16 (3.9)	5 (1.2)	
Renal	8 (1.9)	10 (2.3)	
Median PIM-2, median (IQR)	− 4.58 (− 4.83 to − 3.53)	− 4.55 (− 4.83 to − 3.17)	0.007
Median PRISM-3, median (IQR)	2.00 (0.00–5.00)	3.00 (0.00–7.00)	0.014
Median PRISM-3 POD, median (IQR)	0.51 (0.30–1.08)	0.63 (0.30–1.79)	0.135
Inotrope/vasopressor drip on admission, *n* (%)	63 (15.3)	96 (22.4)	0.008
Ventilated on admission, *n* (%)	65 (15.7)	109 (25.5)	< 0.001
Transport characteristics			
Helicopter (vs. ground), *n* (%)	0 (0)	428 (100)	–
Emergent transport (vs. urgent), *n* (%)	174 (42.1)	134 (31.3)	0.001
Physician present, *n* (%)	82 (19.9)	157 (36.7)	< 0.001
Median mobilization time (h), median (IQR)	0.99 (0.63–1.58)	1.55 (1.17–2.23)	0.005
Median stabilization time (h), median (IQR)	0.52 (0.37–0.75)	0.80 (0.58–1.15)	< 0.001
Median return time (h), median (IQR)	0.50 (0.33–0.75)	0.63 (0.42–1.00)	0.268
Median stabilization + return time (h), median (IQR)	1.08 (0.83–1.53)	1.50 (1.17–2.03)	< 0.001
Median response time (h), median (IQR)	1.65 (0.98–2.65)	2.20 (1.72–3.03)	0.456
Outcomes			
Mortality (within 48 h of hospital admission), *n* (%)	5 (1.2)	8 (1.9)	0.439
Mortality (during hospital admission), *n* (%)	19 (4.6)	35 (8.2)	0.034
Median hospital LOS (d), median (IQR)	4.51 (1.76–10.83)	5.40 (2.43–15.51)	0.001
Median ICU LOS (d), median (IQR)	2.40 (1.24–5.78)	2.97 (1.51–9.45)	0.001

Due to some missing time interval data, time intervals are not additive. *PIM-2* pediatric index of mortality-2, *PRISM-3* pediatric risk of mortality-3, *POD* probability of death, *LOS* length of stay, *ICU* intensive care unit, *IQR* 25–75th interquartile range. “–” none

**Table 4:** Multivariate analyses of the association between transport variables and outcomes

Outcomes	Mortality during admission		Hospital LOS (d)		ICU LOS (d)	
	OR (95% CI)	*P*	Effect (95% CI)	*P*	Effect (95% CI)	*P*
Age (y)	0.97 (0.91 to 1.03)	0.269	− 0.09 (− 0.31 to 0.13)	0.427	− 0.14 (− 0.29 to 0.01)	0.063
Diagnosis		0.185		**0.001**		**0.004**
Respiratory	1.05 (0.13 to 8.41)	0.967	6.36 (0.27 to 12.45)	**0.041**	4.58 (0.46 to 8.69)	**0.029**
Neurologic	0.19 (0.02 to 2.27)	0.190	3.39 (− 2.55 to 9.34)	0.263	1.01 (− 3.01 to 5.02)	0.623
Cardiovascular	1.42 (0.17 to 11.50)	0.744	10.00 (3.19 to 16.81)	**0.004**	7.70 (3.10 to 12.30)	**0.001**
Sepsis	2.04 (0.24 to 17.33)	0.513	11.86 (4.53 to 19.18)	**0.002**	5.14 (0.20 to 10.08)	**0.041**
Gastrointestinal	0.64 (0.06 to 6.79)	0.713	9.06 (1.53 to 16.60)	**0.018**	3.20 (− 1.89 to 8.28)	0.217
Metabolic	0.61 (0.03 to 10.84)	0.733	6.63 (− 1.42 to 14.68)	0.106	2.50 (− 2.93 to 7.94)	0.366
Other	1.09 (0.04 to 28.87)	0.959	3.44 (− 6.19 to 13.07)	0.483	1.31 (− 5.19 to 7.81)	0.693
Hematology/oncology	4.70 (0.46 to 48.42)	0.193	18.20 (8.49 to 27.92)	**< 0.001**	4.46 (− 2.09 to 11.02)	0.182
Renal	0.00 (0.00 to 999)	0.998	12.30 (2.22 to 22.38)	**0.017**	5.54 (− 1.26 to 12.35)	0.110
PIM-2	2.00 (1.40 to 2.86)	**< 0.001**	0.33 (− 1.05 to 1.71)	0.635	0.13 (− 0.80 to 1.06)	0.784
PRISM-3	1.10 (1.03 to 1.17)	**0.002**	− 0.17 (− 0.46 to 0.11)	0.224	− 0.09 (− 0.28 to 0.10)	0.376
Inotrope or vasopressor on admission	1.04 (0.36 to 3.02)	0.936	7.17 (2.91 to 11.44)	**0.001**	5.70 (2.81 to 8.58)	**< 0.001**
Ventilated on admission	0.96 (0.32 to 2.91)	0.946	4.29 (− 0.06 to 8.64)	0.053	4.49 (1.55 to 7.42)	**0.003**
Emergent transport	1.19 (0.50 to 2.85)	0.697	0.80 (− 2.02 to 3.62)	0.577	0.81 (− 1.10 to 2.71)	0.407
Helicopter transport	1.67 (0.71 to 3.90)	0.236	3.24 (0.59 to 5.90)	**0.017**	1.68 (− 0.11 to 3.47)	0.066
Physician presence	0.86 (0.31 to 2.38)	0.777	3.17 (− 0.39 to 6.73)	0.081	1.68 (− 0.72 to 4.08)	0.171
Response time	0.97 (0.84 to 1.11)	0.618	0.15 (− 0.27 to 0.57)	0.486	− 0.02 (− 0.31 to 0.26)	0.880
Stabilization time	0.98 (0.76 to 1.27)	0.889	0.62 (− 0.76 to 2.00)	0.376	0.49 (− 0.44 to 1.43)	0.300
Return time	0.93 (0.57 to 1.51)	0.770	1.13 (− 0.15 to 2.42)	0.083	0.58 (− 0.29 to 1.44)	0.191

Due to some missing time interval data, time intervals are not additive. *P* values with bold letters indicating significance. *PIM-2* pediatric index of mortality-2, *PRISM-3* pediatric risk of mortality-3, *LOS* length of stay, *ICU* intensive care unit, *OR* odds ratio, *CI* confidence interval

## Discussion

Our analysis of 841 pediatric patients revealed that neither physician presence nor response time was significantly associated with mortality, ILOS, or HLOS. We did find that helicopter transports were not significantly associated with mortality or ILOS, but were associated with a longer HLOS. In our retrospective cohort study, we used data from our PCCT team database and electronic medical record to study transport characteristics among a critically ill pediatric patient population with a diverse set of diagnoses. This is the first study to comprehensively investigate the effects of transport team response time, physician presence during transport, and mode of transportation on mortality and LOS among a critically ill pediatric cohort.

Our mortality results are consistent with a study by Sharpe et al. [20] who also found no difference in mortality when adjusting for response time, but this study only analyzed 105 infants less than 29 weeks gestation. Belway et al. [19] found that mortality among adult cardiac patients was not associated with various transport time intervals, but longer response times were associated with a shorter HLOS. The authors speculated that their findings were due to the referring hospital’s ability to stabilize patients before being transported to the specialized unit. These findings are consistent with data from UC Davis Children’s Hospital showing the quality of pre-transport clinical care and close communication with the outside hospital before the PCCT team’s arrival can help improve illness severity scores on admission [24]. We speculate that the effects of prolonged transport times can be mitigated by continued improvements in prehospital care and practice guideline development as well as close communication between accepting and referring hospitals. Some patients, however, may simply need expeditious transport to an accepting hospital for definitive care despite stabilization attempts at a referring hospital or close communication between the referring hospital and the accepting hospital. Given the heterogeneity of our patient cohort in terms of diagnoses, we speculated that certain diagnoses, such as trauma, may be more sensitive to prolonged transport times. To investigate these potential outcome differences between patient subgroups, we attempted a subgroup analysis based on diagnosis, but we could not achieve adequate power.

Herrup et al. [25] surveyed several transport programs and revealed substantial heterogeneity in the subspecialty training level of transport physicians (PICU fellows, neonatology fellows, pediatric emergency fellows, or pediatric anesthesiology fellows) and the years of experience and requirements before joining the team. A survey among transport team members by McCloskey et al. [26] concluded that 46% of respondents felt that a physician was needed to transport critically ill patients. A registry is likely required to track team composition and level of training along with outcomes before any broad conclusions can be made beyond our center. The high level of competency of our PCCT team nurse and respiratory therapist may be the reason for the lack of effect of physician presence on transport. Similar to the physician’s level of training, a formal analysis of the competency of all other transport team members may reveal strategies to further improve safety, outcomes, and cost of high acuity pediatric transports.

Our study did not show a lower mortality or reduced ILOS with helicopter transports. We suspect that pre-transport stabilization, small diagnostic sample sizes, and an overall shorter total transport time relative to a patient’s overall ILOS likely explain these findings. Delgado et al. [27] showed that for helicopter transports to be cost-effective they would need a relative risk reduction in mortality of 15% compared to ground transport when utilizing the cost-effectiveness ratio of less than $100,000 per quality-adjusted life-year gained. Taylor et al. [28] reviewed studies showing helicopter transport programs commonly lack cost-effectiveness. There are no widely used algorithms to determine the indicated or recommended modality of transport among PCCT teams. Transport programs deal with varying local traffic conditions, weather patterns, and team resource limitations, all of which can affect the decision-making process.

Lastly, we speculate that the significant increase in HLOS for helicopter transports compared to ground transports may be due to limitations in vital sign monitoring, altitude and vibration effects, crew resource management during flight, space limitations, and conservative discharge criteria due to potentially long distances for necessary follow-up appointments. These factors are likely minor confounders, thereby less likely to affect ILOS or mortality to the same extent. Without a larger, multi-center study, we caution against using these results to limit this expensive mode of transport among pediatric patients requiring interfacility transport.

Our study has a number of strengths. First, we utilized two standard and commonly calculated illness severity scores, PIM-2, and PRISM-3, while also adjusting for inotrope, vasopressor, and mechanical ventilation use along with various transport time intervals. Second, the heterogeneity of diagnoses and degree of disease severity among our patients allowed us to study a unique population likely to be at the highest risk during interfacility transport. Third, our high rate of helicopter transports due to our hospital’s geographical location, referral base, and traffic congestion allows us to speculate on how helicopter transports can impact medical outcomes. Lastly, we applied multivariate analyses and propensity score modeling as a sensitivity analysis to demonstrate the feasibility of such a study. Conducting a randomized controlled trial evaluating physician presence, mode of transportation, and response time would be challenging and perhaps impossible to conduct ethically or with equipoise.

There are several limitations to our study. Our study may be underpowered to detect a clinically meaningful difference in mortality given that the all-cause mortality during hospitalization was 6.4%. Furthermore, 11.6% of our patients were excluded due to missing data points. Although we attempted to account for confounders by performing multivariate logistic and linear regression analyses as well as propensity score matching, it is possible that we still missed additional confounding effects [29]. We attempted to control for illness severity utilizing PIM-2 and PRISM-3 scores, which, to our knowledge, has not been done in pediatric interfacility transport research. We recognize that PRISM-3 may be an inaccurate reflection of transport illness severity since the score is calculated from data obtained during the first 12 hours of admission. The score could, therefore, be influenced by therapies administered during that time [30]. Future studies utilizing PRISM-4 scores may be more reflective considering this score is calculated using a data range spanning from two hours before admission to four hours after admission [22].

In conclusion, our analysis of 841 pediatric patients revealed that neither physician presence nor response time was significantly associated with mortality, ILOS or HLOS. We did find that helicopter transports were not significantly associated with mortality or ILOS, but were associated with a longer HLOS. Despite the limitations of a single-center study, our analysis provides a framework for examining transport workforce needs and helps guide further studies using large PCCT databases to further characterize the impact of time intervals, mode of transport, and physician presence.
